# The antiarrhythmic compound efsevin directly modulates voltage‐dependent anion channel 2 by binding to its inner wall and enhancing mitochondrial Ca^2+^ uptake

**DOI:** 10.1111/bph.15022

**Published:** 2020-03-25

**Authors:** Fabiola Wilting, Robin Kopp, Philip A. Gurnev, Anna Schedel, Nathan J. Dupper, Ohyun Kwon, Annette Nicke, Thomas Gudermann, Johann Schredelseker

**Affiliations:** ^1^ Walther Straub Institute of Pharmacology and Toxicology, Faculty of Medicine LMU Munich Munich Germany; ^2^ Section on Molecular Transport, Eunice Kennedy Shriver National Institute of Child Health and Human Development National Institutes of Health Bethesda Maryland; ^3^ Department of Chemistry and Biochemistry University of California Los Angeles Los Angeles California; ^4^ Deutsches Zentrum für Herz‐Kreislauf‐Forschung (DZHK) Partner Site Munich Heart Alliance (MHA) Munich Germany

## Abstract

**Background and Purpose:**

The synthetic compound efsevin was recently identified to suppress arrhythmogenesis in models of cardiac arrhythmia, making it a promising candidate for antiarrhythmic therapy. Its activity was shown to be dependent on the voltage‐dependent anion channel 2 (VDAC2) in the outer mitochondrial membrane. Here, we investigated the molecular mechanism of the efsevin–VDAC2 interaction.

**Experimental Approach:**

To evaluate the functional interaction of efsevin and VDAC2, we measured currents through recombinant VDAC2 in planar lipid bilayers. Using molecular ligand‐protein docking and mutational analysis, we identified the efsevin binding site on VDAC2. Finally, physiological consequences of the efsevin‐induced modulation of VDAC2 were analysed in HL‐1 cardiomyocytes.

**Key Results:**

In lipid bilayers, efsevin reduced VDAC2 conductance and shifted the channel's open probability towards less anion‐selective closed states. Efsevin binds to a binding pocket formed by the inner channel wall and the pore‐lining N‐terminal α‐helix. Exchange of amino acids N207, K236 and N238 within this pocket for alanines abolished the channel's efsevin‐responsiveness. Upon heterologous expression in HL‐1 cardiomyocytes, both channels, wild‐type VDAC2 and the efsevin‐insensitive VDAC2^AAA^ restored mitochondrial Ca^2+^ uptake, but only wild‐type VDAC2 was sensitive to efsevin.

**Conclusion and Implications:**

In summary, our data indicate a direct interaction of efsevin with VDAC2 inside the channel pore that leads to modified gating and results in enhanced SR‐mitochondria Ca^2+^ transfer. This study sheds new light on the function of VDAC2 and provides a basis for structure‐aided chemical optimization of efsevin.

**Abbreviations**
CPVT: catecholaminergic polymorphic ventricular tachycardia
DPhPC: 1,2‐diphytanoyl‐sn‐glycero‐3‐phosphatidylcholine
hVDAC2: human voltage‐dependent anion channel 2
IRES: internal ribosome entry site
MCU: mitochondrial calcium uniporter
MOI: multiplicity of infection
mVDAC2: mouse voltage‐dependent anion channel 2
OMM: outer mitochondrial membrane
P_O_: open probability
RuR: ruthenium red
shRNA: short hairpin RNA
SR: sarcoplasmic reticulum
VDAC: voltage‐dependent anion channel
zVDAC2: zebrafish voltage‐dependent anion channel 2

**What is already known**

- The synthetic compound efsevin suppresses arrhythmogenesis in cardiac arrhythmia models.
- Efsevin binds to the mitochondrial voltage‐dependent anion channel anion channel 2 (VDAC2).

**What this study adds**

- Efsevin binds into a pocket formed by the channel wall and the pore‐lining helix.
- Efsevin facilitates VDAC2 gating into a less anion‐selective state and enhances Ca^2+^ flux.

**What is the clinical significance**

- Identification of mode of action and binding pocket allows for future structure aided‐drug optimization.

## 1 INTRODUCTION

Cardiovascular diseases represent the primary cause of death and hospitalization worldwide (Benjamin et al., 2018). Especially, Cardiac arrhythmias are difficult to treat due to major side effects of common antiarrhythmic drugs. It is thus a major focus of cardiovascular research to identify novel, safer therapies for cardiac arrhythmia.

Using a chemical suppressor screen on the zebrafish cardiac arrhythmia model *tremblor* (Ebert et al., 2005; Langenbacher et al., 2005), we have previously identified the synthetic compound efsevin, a dihydropyrrole carboxylic ester compound, which potently restored rhythmic cardiac contractions in otherwise fibrillating zebrafish embryonic hearts (Shimizu et al., 2015). We further demonstrated efficacy of efsevin in translational models for catecholaminergic polymorphic ventricular tachycardia (CPVT; Schweitzer et al., 2017). Here, efsevin reduced episodes of tachycardia in vivo in CPVT mice and suppressed arrhythmogenic events in induced pluripotent stem cell‐derived cardiomyocytes from a CPVT patient (Schweitzer et al., 2017). These findings make efsevin a promising lead structure for human antiarrhythmic therapy.

In a pull‐down assay with immobilized efsevin, the outer mitochondrial membrane (OMM) voltage‐dependent anion channel 2 (VDAC2) was identified as the primary molecular target of efsevin (Shimizu et al., 2015). Voltage‐dependent anion channels are large pore‐forming proteins in the outer mitochondrial membrane. They represent the main pathway for ions and metabolites over the outer mitochondrial membrane. Three isoforms of voltage‐dependent anion channels are expressed in vertebrates out of which VDAC2 was described to have a specific role in the heart. While a global knockout of VDAC2 in mice is embryonically lethal (Cheng, Sheiko, Fisher, Craigen, & Korsmeyer, 2003), a conditional heart‐specific VDAC2 knockout mouse was reported to develop post‐natal cardiac defects and to die shortly after birth (Raghavan, Sheiko, Graham, & Craigen, 2012). In cardiomyocytes, VDAC2 was described to interact with the ryanodine receptor (RyR; Min et al., 2012) and to modulate cytosolic Ca^2+^ signals (Shimizu et al., 2015; Subedi et al., 2011). In arrhythmic *tremblor* zebrafish embryos, efsevin induced a restoration of rhythmic cardiac contractions. Transient knock‐down of VDAC2 abolished the efsevin induced phenotype restoration while overexpression of VDAC2 recovered it. In cultured cells, efsevin enhanced uptake of Ca^2+^ into mitochondria. A direct link between the enhanced mitochondrial Ca^2+^ uptake and efsevin's anti‐arrhythmic properties was established by two lines of experiments: (a) Pharmacological inactivation of mitochondrial Ca^2+^ uptake by Ru360, an inhibitor of the mitochondrial Ca^2+^ uniporter (MCU), abolished efsevin's protective effect in CPVT cardiomyocytes and (b) kaempferol, an activator of the mitochondrial Ca^2+^ uniporter, reduced arrhythmogenic Ca^2+^ signals comparable to efsevin (Schweitzer et al., 2017).

However, biophysical and structural determinants of the efsevin–VDAC2 interaction have remained unexplored. It is still unclear whether efsevin directly interacts with the VDAC2 peptide and thereby modulates the electrophysiological properties of the channel or if the observed effects require a yet unidentified protein partner. To address this issue, we expressed and purified recombinant zebrafish VDAC2 (zVDAC2) protein and inserted it into planar lipid bilayers. We found a pronounced effect of efsevin on channel gating and opening probability, indicating a direct effect of efsevin on the channel. To analyse the structural basis of the efsevin VDAC2 interaction, we performed computational protein‐ligand docking using the crystal structure of zVDAC2 (Schredelseker et al., 2014). VDAC2 is formed by a barrel‐like structure consisting of 19 antiparallel β‐sheets (β‐sheets 1‐19) and an N‐terminal α‐helix lining the inner channel wall (Schredelseker et al., 2014). We identified an efsevin binding site located in a groove between the inner channel wall and the pore‐lining α‐helix and formed by hydrogen bonds and hydrophobic interactions between efsevin and the zVDAC2 peptide. Replacement of three residues (N207, K236 and N238) from this binding site with alanines (zVDAC2^AAA^) resulted in a complete loss of efsevin sensitivity. To evaluate the physiological consequence of the observed efsevin‐induced biophysical changes in VDAC2, we heterologously expressed wild‐type zVDAC2 and the efsevin‐insensitive zVDAC2^AAA^ mutant in cultured HL‐1 cardiomyocytes. We demonstrate that the observed changes in zVDAC2 electrophysiology translate into enhanced mitochondrial Ca^2+^ uptake and thereby explain the antiarrhythmic effect of efsevin. Our data provide novel insights into VDAC2 function and provide a basis for structure‐aided chemical optimization of efsevin as a lead structure for the development of novel antiarrhythmic drugs.

## 2 METHODS

### 2.1 Expression and purification of zVDAC2

Expression and purification of zVDAC2 were performed as previously described (Schredelseker et al., 2014) with minor modifications: Histidine‐tagged zVDAC2 was purified on an Äkta pure chromatography system using a HisPrep FF 16/10 column. After refolding dialysis, size exclusion chromatography was performed on a HiLoad 16/600 Superdex200 pg column equilibrated with 150‐mM NaCl, 1‐mM DTT, 0.1% LDAO, and 20‐mm Tris‐HCl (pH = 8.0). Integrity of the purified zVDAC2 was confirmed by SDS‐PAGE analysis on a 12% gel.

### 2.2 Planar lipid bilayer recordings

Painted planar lipid bilayers were formed on an Ionovation Bilayer Explorer lipid bilayer set‐up across a 120‐μm diameter opening with 1,2‐diphytanoyl‐sn‐glycero‐3‐phosphatidylcholine (DPhPC) dissolved in *n*‐decane at 12.5 mg·ml^−1^. Both chambers were filled with 1‐M KCl, 5‐mM CaCl_2_, and 10‐mM Tris‐HCl (pH = 7.2) To facilitate insertion, purified zVDAC2 was inserted into lipidic bicelles (Ujwal, 2012). After formation of a stable bilayer, zVDAC2 containing bicelles were added to the cis chamber. After insertion of a channel, the membrane was clamped to 0 mV, and 10 s pulses to test potentials from −60 mV to +60 mV were applied. The signal was sampled at 10 kHz and filtered at 2 kHz. Efsevin (50‐μM stock in recording solution) was added to the cis chamber to a final concentration of 8 μM. Data analysis was performed using Nest‐o‐Patch (Dr. V. Nesterov, https://sourceforge.netq/projects/nestopatch/).

zVDAC2 ion selectivity was measured using folded planar membranes formed from opposition of two monolayers made of 5 mg·ml^−1^ solution of DPhPC in pentane, as described (Rostovtseva, Gurnev, Chen, & Bezrukov, 2012). Recordings were performed in the buffer described above. Channel insertion was achieved by adding zVDAC2 in a 2.5% Triton X‐100 solution to the cis compartment while stirring. After single channel was inserted in symmetrical solutions, the cis side was perfused with Tris‐HCl (pH 7.2) buffer solution to achieve 0.2‐M KCl. The exact KCl concentration of 0.2 M in the cis compartment after perfusion was verified at the end of each selectivity experiment using a conductivity meter CDM230 (Radiometer analytical). Ion selectivity of zVDAC2 conductance states was calculated from the reversal potential (*V*_rev_). Permeability ratio between Cl^−^, and K^+^, *I* ^*−*^ */I* ^*+*^, was calculated according to the Goldman–Hodgkin–Katz equation (Hille, 2001):

$$I^{-}/I^{+}=\left(1-\frac{V_{\mathrm{rev}}}{\frac{k_{B}T}{e}\cdot \ln\frac{a_{\text{trans}}}{a_{\mathrm{cis}}}}\right)\cdot {\left(1+\frac{V_{\mathrm{rev}}}{\frac{k_{B}T}{e}\cdot \ln\frac{a_{\text{trans}}}{a_{\mathrm{cis}}}}\right)}^{-1},$$

where *k*_*B*_, *T*, and *e* have their usual meaning of Boltzmann constant, absolute temperature, and electron charge, and KCl solution activities were *a*_cis_ = 0.144 for 0.2‐M KCl and *a*_trans_ = 0.604 for 1‐M KCl, respectively (Lide, 2006).

### 2.3 Molecular docking

The three‐dimensional (3D) structure of efsevin was generated using MarvinSketch (v15.1.9, ChemAxon) and saved in the .pdb format. The crystal structure of zVDAC2 was obtained from the Research Collaboratory for Structural Bioinformatics Protein Data Bank (PDB ID: 4BUM). Gasteiger charges and hydrogens were added to protein and ligand using AutoDockTools (v1.5.6; Morris et al., 2009), and AutoDock Vina was used to perform the molecular docking simulations (Trott & Olson, 2010). The term “independent docking experiment” used in this text refers to independent software runs of AutoDock Vina. Each run starts from a random conformation that is independent from the previous one and yields nine likely conformations. To determine the best hypothetical binding mode of efsevin on zVDAC2, the flexible form of efsevin was first docked to the rigid zVDAC2 structure. After 15 iterative dockings with a grid centred to ensure coverage of the entire channel, analysis of 135 binding conformations showed that the binding site with the lowest binding energy was found in the region between the inner channel wall and the highly flexible N‐terminal α‐helix. Since zVDAC2 might undergo conformational changes upon binding of efsevin, the side chains facing the potential binding site were kept flexible for subsequent dockings (N19, Y22, F24, N207, R218, K236, N238, L242 and L262). The grid box was narrowed to 24 Å × 20 Å × 20 Å to cover this region. After additional 15 iterative dockings with flexible side chains, the best binding poses were selected based on the predicted binding affinities. Interactions were analysed with LigPlot+ (Laskowski & Swindells, 2011).

### 2.4 Molecular cloning

To introduce N207A, K236A and N238A into pQE60‐zVDAC2 for recombinant expression and purification construct pQE60‐zVDAC2^AAA^ was created by two mutagenesis PCR reactions using pQE60‐zVDAC2 (Schredelseker et al., 2014) as a template. The two PCR fragments were fused by overlap extension PCR and the resulting product was ligated into pQE60‐zVDAC2 with PstI and NheI.

For the creation of pCClc‐CMV‐zVDAC2‐IRES‐nlsEGFP for virus production and following transduction of zVDAC2 into HL‐1 cells, eGFP with a nuclear localization signal (nls‐eGFP) together with an internal ribosome entry site (IRES) was PCR‐amplified from p3E‐IRES‐nlsEGFPpA (Kwan et al., 2007) and subcloned into pCS2+ containing the zVDAC2 open reading frame (Shimizu et al., 2015). The zVDAC2‐IRES‐nlsEGFP construct was fused into pCCLc‐CMV using the In‐Fusion HD Cloning Kit (TaKaRa).

For the creation of pCClc‐CMV‐zVDAC2^AAA^‐IRES‐nlsEGFP, zVDAC2 was exchanged for zVDAC2^AAA^ in pCS2+‐zVDAC2 (Shimizu et al., 2015) by PCR amplification of the zVDAC2^AAA^ open reading frame from PQE60‐zVDAC2^AAA^ following ligation with BamHI and ClaI. The open reading frame of zVDAC2^AAA^ was PCR‐amplified from pCS2+‐zVDAC2^AAA^ fused to the IRES PCR‐amplified from pCClc‐CMV‐zVDAC2‐IRES‐nlsEGFP by SOE PCR and the resulting fragment was fused into pCClc‐CMV‐zVDAC2‐IRES‐nlsGFP using the In‐Fusion HD Cloning Kit (TaKaRa).

### 2.5 Production of lentiviruses

Recombinant lentiviruses were produced in HEK 293T cells. Briefly, cells at 40% confluency were cotransfected with pCCLc‐CMV‐zVDAC2‐IRES‐nlsEGFP or pCCLc‐CMV‐zVDAC2^AAA^‐IRES‐nlsEGFP together with pCMVΔ8.91 (coding for gag, pol, rev) and pCAGGS‐VSV‐G at a 5:5:1 ratio using TransIT®‐293 (Mirus). Cells were induced with 10‐mM sodium butyrate on the following day. The supernatant containing the virus was collected on Day 3 after transfection, filtered through a 0.45‐μm syringe filter, and stored at −80°C. Viral titres were determined using the Lenti‐X™ qRT‐PCR Titration Kit (Takara).

### 2.6 HL‐1 culture and creation of cell lines

HL‐1 cells were cultured as described previously (Claycomb et al., 1998). Briefly, HL‐1 cells were grown on fibronectin (0.02%) / gelatin (10 μg·ml^−1^) coated flasks in Claycomb medium supplemented with 10% FBS, 100 μg·ml^−1^ penicillin/streptomycin, 0.1‐mM norepinephrine, and 2‐mM l‐glutamine. Stable knockdown of the endogenous mVDAC2 in HL‐1 cardiomyocytes was performed by lentiviral transduction with shLenti2.4G‐mVDAC2 or shLenti2.4G‐Ctrl as described previously (Subedi et al., 2011) In brief, HL‐1 cells were transduced in serum‐free Claycomb medium with lentivirus at a multiplicity of infection (MOI) of 30 in the presence of 8 μg·ml^−1^ polybrene. Three days after transduction, cells were selected with 2 μg·ml^−1^ puromycin for 10 days and cultured for additional three passages. For overexpression of zVDAC2 and zVDAC2^AAA^, transduction was performed as described above using a MOI of 25. After 4 days, nlsEGFP‐positive cells were selected by FACS sorting on a BD FACS Aria III.

### 2.7 SR‐mitochondria Ca^2+^ transfer

Fluorescence‐based measurements of mitochondrial Ca^2+^ were performed as previously described (Schweitzer et al., 2017). In brief, HL‐1 cells (RRID:CVCL_0303) were plated in 96‐well plates 1 day prior to the experiment. To monitor mitochondrial Ca^2+^, cells were stained with Rhod‐2, AM (Thermo Fisher, Darmstadt, Germany) and permeabilized using digitonin. Mitochondrial Ca^2+^ was constantly monitored in a fluorescence plate reader (Infinite® 200 PRO multimode reader, Tecan, Maennedorf, Switzerland), and SR Ca^2+^ release was induced by superfusion with 10‐mM caffeine.

### 2.8 Metabolic stability assay

Efsevin (10 mM in DMSO) was co‐incubated with human liver microsomes at 37°C at an initial concentration of 1 μM. The reaction was initiated by addition of 1‐mM NADPH; 0, 5, 10, 30, and 60 minutes after starting the reaction, small aliquots were transferred into ice‐cold acetonitrile and centrifuged at 16000× *g* for 10 min. Supernatants were analysed by LC–MS/MS. Experiments were performed as contract research at 3D BioOptima Co., Ltd., Suzhuo, China.

### 2.9 Data and statistical analysis

The data and statistical analysis comply with the recommendations of the *British Journal of Pharmacology* on experimental design and analysis in pharmacology (Curtis et al., 2018). zVDAC2 channels were inserted into lipid bilayers and measured before and after addition of efsevin in recording solution, thus leading groups of equal size. In some instances, membranes ruptured during the recording explaining smaller sample size for recordings with efsevin. The recording before efsevin addition served as a control. Since efsevin was the only drug investigated and was added in measuring solution, randomization and blinding were not applied. All traces were equally analysed for predefined parameters like average current or open state to exclude operators bias. For Ca^2+^ uptake experiments on HL‐1 cardiomyocytes, wells were randomly assigned to control, efsevin and ruthenium red (RuR) group to generate groups of equal size. Wells were microscopically inspected after the experiment, and those in which cells have detached were excluded from the analysis. No data transformation or outlier removal was performed, and all traces were equally analysed for maximum response by a predefined script to exclude operators bias. Since nature and magnitude of the effects of efsevin on zVDAC2 were unknown before the experiment and not predictable, a sample size evaluation was not feasible.

Statistical analysis was only performed for sample sizes >5 from independent experiments (i.e. individual insertions in lipid bilayers, individual runs of computational docking, and individual cultures of HL‐1 cardiomyocytes), and the obtained data were used for statistical analysis without normalization and outlier removal. Statistical analysis was performed using Prism 7 (GraphPad Software, San Diego, USA). Data are presented as mean ± *SEM* of *n* individual experiments (no technical replicates). Normality of data was determined by Shapiro–Wilk test. Tests for statistical significance were conducted as indicated and where unpaired student's t‐test for comparison of two groups with normal distribution of data, Mann Whitney U test for comparison of two groups with non‐normal distribution of data, and Kruskal‐Wallis test with Dunn's post hoc test for multigroup comparison with non‐normal data distribution. Statistical significance of *P* < .05 is indicated as *.

### 2.10 Materials

Efsevin was synthesized as described before (Henry et al., 2014). Protein purification was performed on an Äkta pure, and all Äkta material was purchased from GE Healthcare, Munich, Germany; LDAO was obtained from VWR, Darmstadt, Germany. The lipid bilayer set‐up and all consumables including 1,2‐diphytanoyl‐sn‐glycero‐3‐phosphatidylcholine (DPhPC) were purchased from Ionovation, Osnabrück, Germany. Ruthenium red and caffeine used for the HL‐1 Ca^2+^ uptake assay were purchased from Sigma‐Aldrich (Munich, Germany). Plasmids pCCLc‐CMV, pCMVΔ8.91 and pCAGGS‐VSV‐G were obtained as a gift from Dr. Donald Kohn, University of California Los Angeles, USA; plasmid shLenti2.4G‐mVDAC2 and shLenti2.4G‐Ctrl were generously provided by Dr. Yeon Soo Kim from Inje University, Gimhae, South Korea. HL‐1 cardiomyocytes were obtained as a gift from Dr. William Claycomb, Louisiana State University, New Orleans, USA; Claycomb medium and all cell culture supplies were obtained from Sigma‐Aldrich (Munich, Germany).

### 2.11 Nomenclature of targets and ligands

Key protein targets and ligands in this article are hyperlinked to corresponding entries in http://www.guidetopharmacology.org, the common portal for data from the IUPHAR/BPS Guide to PHARMACOLOGY (Harding et al., 2018), PHARMACOLOGY.

## 3 RESULTS

### 3.1 Efsevin reduces currents through zVDAC2

**FIGURE 1:**
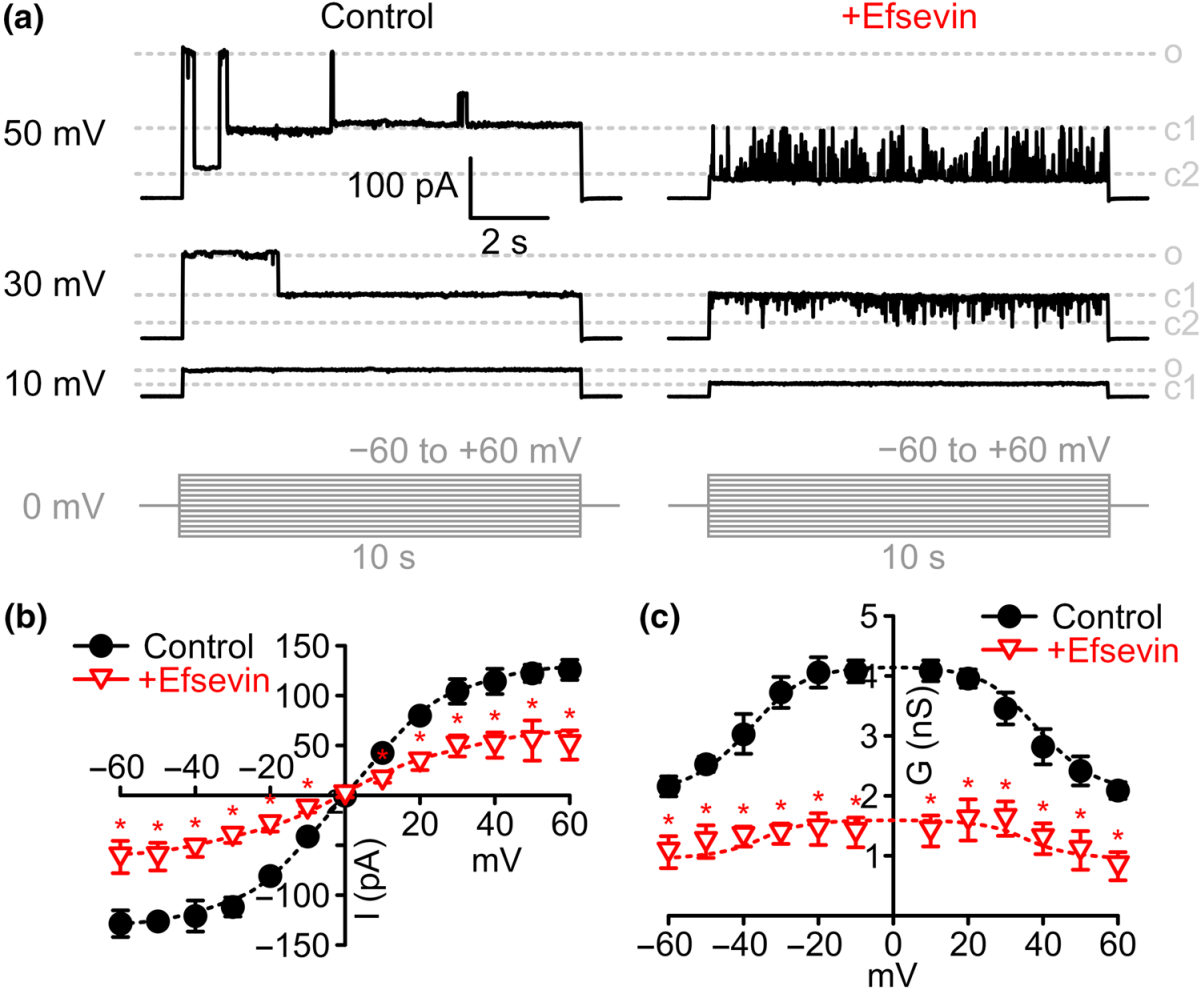
Effects of efsevin on zVDAC2 currents in planar lipid bilayers. (a) Typical current recordings from zVDAC2 inserted into painted planar DPhPC lipid bilayers in 1‐M KCl in response to 10 s test pulses from 0 to 10, 40, and 50 mV, respectively, under control conditions (left) and after addition of 8‐μM efsevin (right) to the same channel. Gating between three major states open (o), closed1 (c1) and closed2 (c2) and few subconductance states can be observed (pulse protocol in shown in grey). (b) Current–voltage relationship of average zVDAC2 currents before (*n* = 9 individual channels, black circles) and after addition of efsevin (*n* = 6 individual channels, red triangles, Unpaired Student's *t‐*test). (c) Conductance–voltage relationship of zVDAC2 before (*n* = 9 individual channels, black circles) and after addition of efsevin (*n* = 6 individual channels, red triangles, Unpaired Student's *t‐*test)

Recombinant zVDAC2 protein was purified from *Escherichia coli* as previously described (Schredelseker et al., 2014) and inserted into diphytanoylphosphatidylcholine (DPhPC) planar lipid bilayers. Currents were measured in response to 10 s test pulses to potentials from −60 to +60 mV in 1‐M KCl. As shown in Figure 1a, typical average currents of 41 ± 3pA were recorded at 10 mV, where the channel almost exclusively resides in its open state (Colombini, 1989; Schredelseker et al., 2014). VDAC‐typical flattening of the current–voltage relationship (*I*–*V* curve) was observed starting at approximately ±30 mV (Figure 1b), where the channel starts gating until finally being preferentially in its closed state at potentials above +50 mV or below −50 mV (Figure 1a,b). The observed *I*–*V* curve for zVDAC2 translates into a bell‐shaped conductance–voltage relationship that is characteristic of VDACs (Figure 1c). Strikingly, addition of 8‐μM efsevin destabilized the channel's high conductance state and dramatically reduced currents through zVDAC2 by about 50% across all voltages.

### 3.2 Efsevin reduces zVDAC2 open probability and shifts the channel towards closed states

**FIGURE 2:**
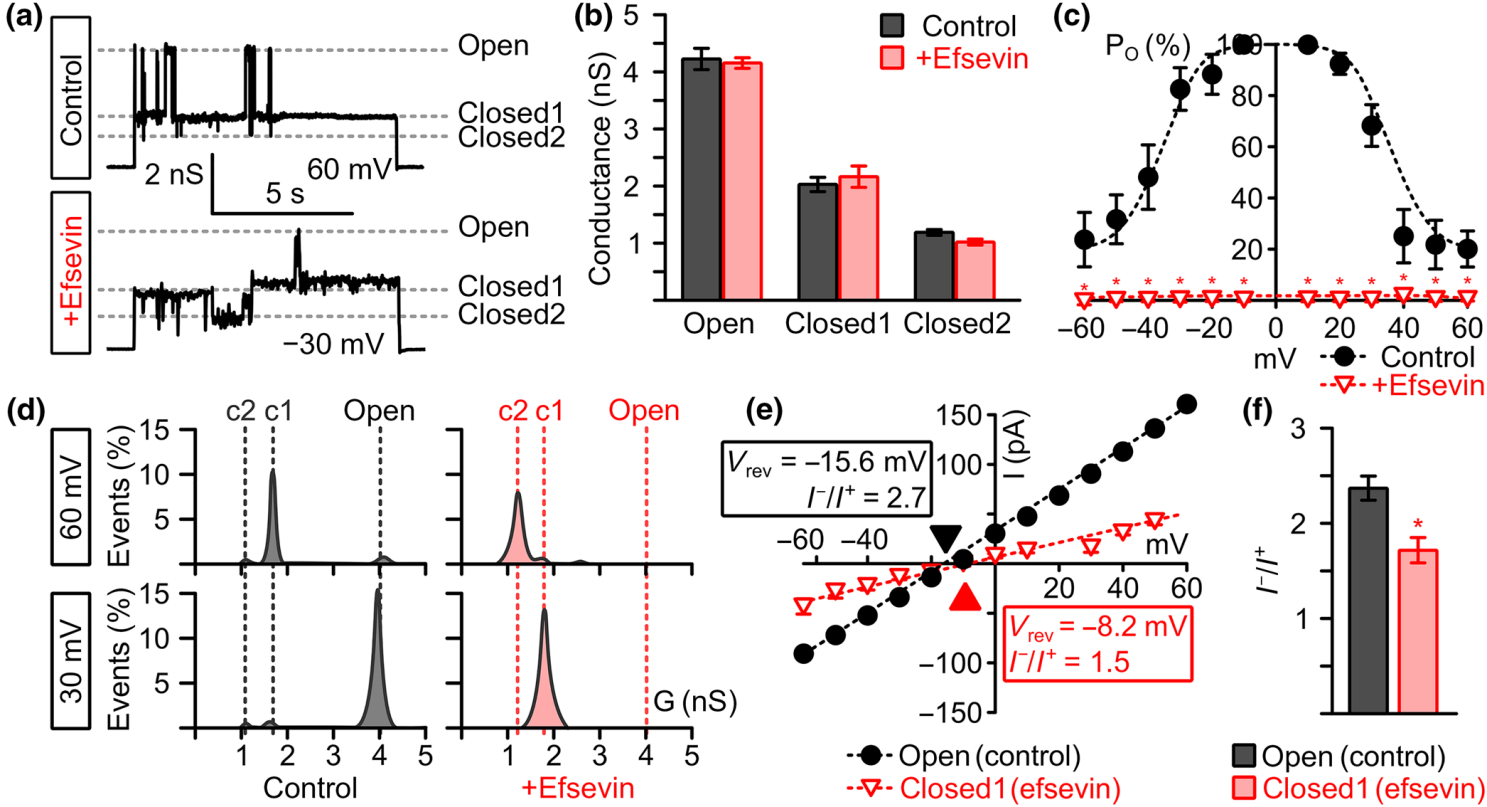
Effects of efsevin on zVDAC2 conductance and open probability (P_O_). (a) Representative recordings of zVDAC2 in a painted lipid bilayer under control conditions (upper trace) and after addition of 8‐μM efsevin (lower trace) at potentials where typical gating behaviour between the three states is observed, that is, very low/high potentials under control conditions and moderate potentials after addition of efsevin. Currents through the open and two distinct closed states of the channel are indicated by dashed lines. (b) Addition of 8‐μM efsevin does not change the conductance of the three distinct conductance states of zVDAC2 (*n* = 12 individual channels for control and 6 individual channels after addition of efsevin). (c) Analysis of the open probability (P_O_) of zVDAC2 shows a significant shift of the channel towards the closed states across all voltages after addition of efsevin (*n* = 12 for control and *n* = 6 for efsevin, Mann–Whitney *U* test). (d) Representative conductance histograms from recordings of zVDAC2 with and without efsevin at 30 mV and 60 mV show a shift of the channel from the classical open state to the closed states. (e,f) Current–voltage plots for the open and the efsevin‐induced closed state 1 obtained from zVDAC2 in a folded lipid bilayer using a 0.2‐M to 1‐M KCl gradient reveal a shift in the reversal potential (*V*_rev_). (f) Quantitative analysis of selectivity measurements reveals a significant reduction in anion selectivity for the efsevin‐induced closed state 1 (*n* = 6 for control, *n* = 5 for efsevin, Unpaired Student's *t‐* test)

Since efsevin profoundly reduced average zVDAC2 conductance, we further analysed the biophysical properties of zVDAC2 single channel currents to dissect the effects of efsevin on single channel conductance versus open probability of the channel. VDAC has been shown to undergo voltage‐dependent gating between an anion‐selective high‐conductance state, referred to as the classical open state and several cation‐selective low‐conductance states, referred to as closed states (Colombini, 1989; Guardiani et al., 2018; Menzel et al., 2009; Mertins et al., 2012). Under control conditions, we observed gating of the channel between the classical open state with a conductance 4.2 ± 0.2 ns and two closed states with conductances of 2.0 ± 0.1 ns for closed state 1 and 1.2 ± 0.04 ns for closed state 2 (Figure 2a,b). Efsevin shifts the channel mainly into closed states 1 and 2 with only a few scarce openings of the channel into the open state, already at low potentials. Conductances of the three states remained comparable to control conditions (open: 4.1 ± 0.1 ns; closed1: 2.1 ± 0.2 ns; closed2: 1.0 ± 0.04 ns; Figure 2b). The shift towards the closed states becomes most obvious when plotting the open probability (P_O_) of the channel against voltage (Figure 2c). Interestingly, at the physiologically relevant low potentials of ±30 mV (Lemeshko, 2006; Porcelli et al., 2005), the apo‐form of the channel resides mainly in the open state, while the efsevin‐bound form is preferentially closed (Figure 2d). The VDAC closed states were previously defined as more cation‐selective compared to the open state (Tan & Colombini, 2007; Zachariae et al., 2012). We therefore measured the ion selectivity of the dominant states, the open state of the apo‐form and closed state 1 of the efsevin‐bound channel using salt gradient conditions (0.2‐M [cis] vs. 1‐M [trans] KCl) and found a change in the Cl^−^ to K^+^ permeability ratio *I* ^−^/*I* ^+^ from 2.38 ± 0.13 for the open state to 1.71 ± 0.14 for the efsevin‐bound closed state 1 (Figure 2e,f). In conclusion, our data indicate that efsevin binding facilitates channel closure and induces a shift of zVDAC2 towards the less anion‐selective low conductance states.

### 3.3 Molecular docking reveals an efsevin binding site between the inner channel wall and the pore‐lining N‐terminal α‐helix

**FIGURE 3:**
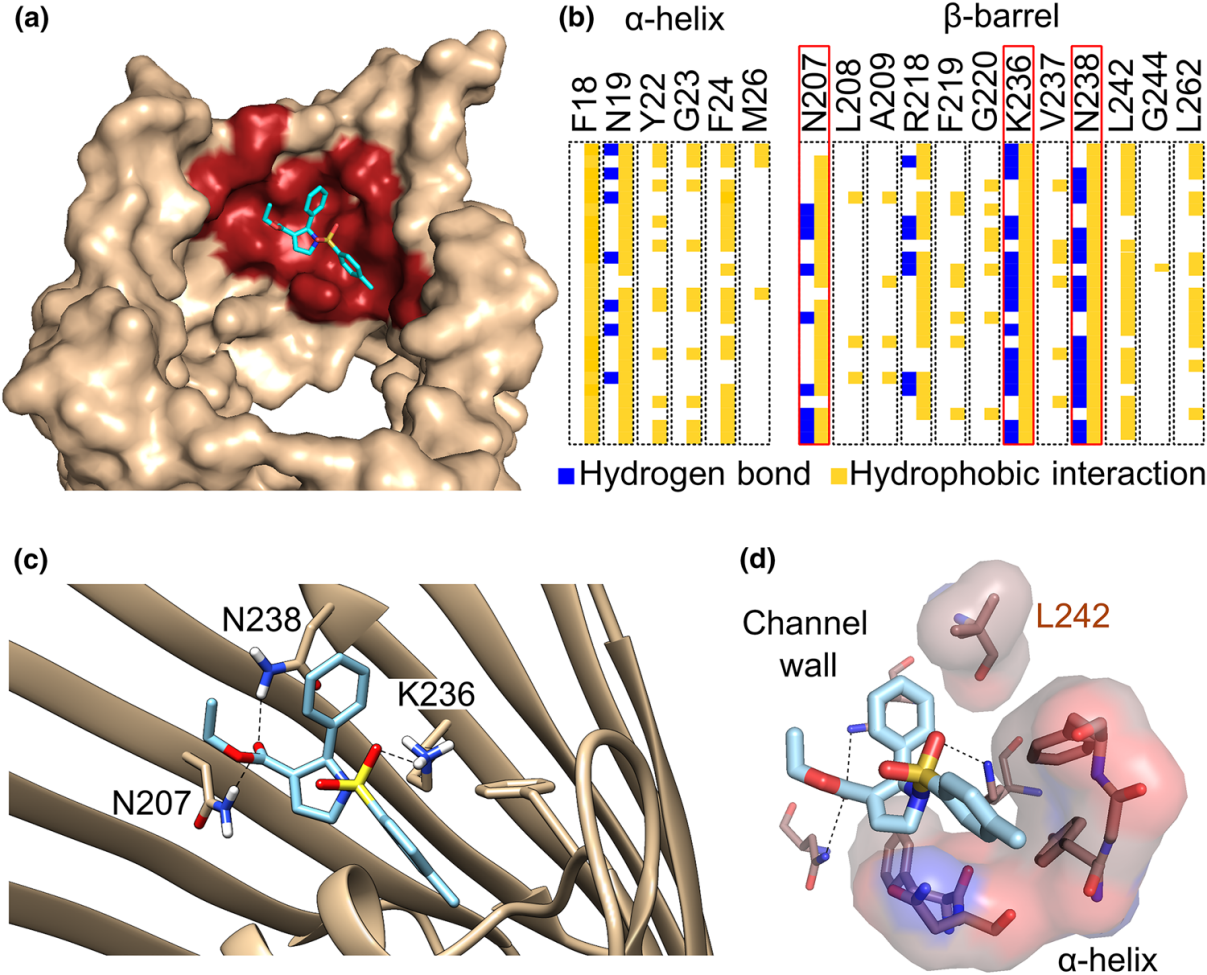
Predicted binding site of efsevin on zVDAC2 obtained by molecular docking. (a) Side view of the channel (pdb: 4bum, light brown) displays a binding pocket (red) for efsevin (light blue) located in the interspace between the inner channel wall and the N‐terminal pore‐lining α‐helix. (b) Analysis of 25 different molecular dockings (rows) reveals interactions between efsevin and 18 amino acid rests of zVDAC2 (columns) through hydrogen bonds (blue) and hydrophobic interactions (yellow). Residues with the most interactions (N207 in β‐sheet 14, K236, and N238 in β‐sheet 16 of the barrel) are boxed in red. (c) Detailed view of one representative docking with efsevin binding to the channel through hydrogen bonds between the most prominent amino acids highlighted in (b) and efsevin. (d) Surface representation of residues forming hydrophobic interactions in the conformation shown in (c) reveals a cavity that accommodates the *p*‐tolyl group of efsevin on the hinge of the flexible N‐terminal α‐helix. Nitrogen is shown in blue, oxygen in red, and sulfur in yellow

To identify the interaction between efsevin and zVDAC2 at the molecular level, we performed protein‐ligand docking using AutoDock Vina (Trott & Olson, 2010). By docking the flexible form of efsevin into the rigid crystal structure of zVDAC2 (Schredelseker et al., 2014), a binding pocket formed between the inner channel wall and the N‐terminal α‐helix of zVDAC2 repeatedly showed the lowest binding energies in 15 independent dockings yielding 135 conformations (Figures 3a and S1). To further investigate molecular interactions between zVDAC2 and efsevin within this pocket, we performed subsequent docking simulations in which residues facing this binding site were made flexible. We identified multiple conformations with binding affinities below −8.0 kcal·mol^−1^ in which the dihydropyrrole of the efsevin core with the ethoxycarbonyl and phenyl substituents was always interacting with residues in the channel wall, while the *p*‐tolyl substituent of the sulphonamide group faces the lumen of the pore and interacts with the hinge region of the N‐terminal α‐helix. Interactions of efsevin with zVDAC2 were identified with residues F18, N19, Y22, G23, F24 and M26 in the N‐terminal α‐helix and with residues N207, L208, A209, R218, F219, G220, K236, V237, N238, L242, G244 and L262 in the β‐barrel, whereof asparagine 207, lysine 236 and asparagine 238 were the most prominent interaction partners. Hydrogen bonds and hydrophobic interactions with these three amino acids were present in the majority of conformations (Figure 3b). Figure 3c,d shows one representative conformation in which efsevin is held in place by N207, K236 and N238 in the channel wall and interacts with the α‐helix through hydrophobic interactions with residues F18, N19, Y22, G23 and F24. To finally investigate translatability of the docking results to the human channel, we created a model of human VDAC2 (hVDAC2) by homology modelling using SWISS‐MODEL (Waterhouse et al., 2018) since no experimentally determined structure for hVDAC2 is currently available. In eight out of 10 independent docking experiments, the conformation with the lowest binding energy was found in the same binding pocket like in zVDAC2 indicating that this binding pocket is conserved among species (Figure S2).

### 3.4 Elimination of residues N207, K236 and N238 abolishes the efsevin sensitivity of zVDAC2

**FIGURE 4:**
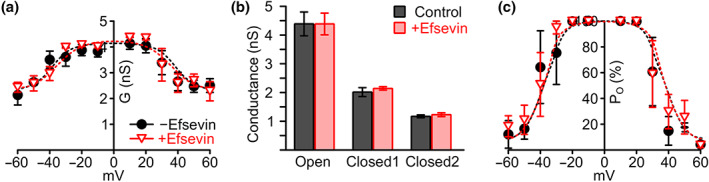
Effects of efsevin on conductance, and open probability (P_O_) of zVDAC2^AAA^ in lipid bilayers. (a) Conductance–voltage relation of zVDAC2^AAA^ in painted bilayer recordings reveals a comparable shape before and after addition of 8‐μM efsevin. (b) No differences in single channel conductance for open and closed states of zVDAC2^AAA^ were observed after addition of efsevin. (c) Finally, in zVDAC2^AAA^, efsevin was unable to induce the reduction of P_O_ observed for wild‐type zVDAC2 (*n* = 4 individual channels for control and 3 individual channels after addition of efsevin)

To confirm the predicted efsevin binding site by mutational analysis, we created a zVDAC2 mutant in which residues N207, K236 and N238 were substituted by alanine residues, zVDAC2^N207A/K236A/N238A^ (zVDAC2^AAA^). Lipid bilayer recordings of this mutant showed that it forms a functional channel with a similar conductance–voltage relationship, single channel conductance and P_O_ compared to wild‐type zVDAC2 (Figure S3). Most strikingly however, the channel was insensitive to efsevin, demonstrated by comparable values for the conductance–voltage relationship, single channel conductance and P_O_ before and after addition of efsevin (Figure 4).

### 3.5 Efsevin mediates SR‐mitochondria Ca^2+^ transfer by binding to the N207/K236/N238 binding site

**FIGURE 5:**
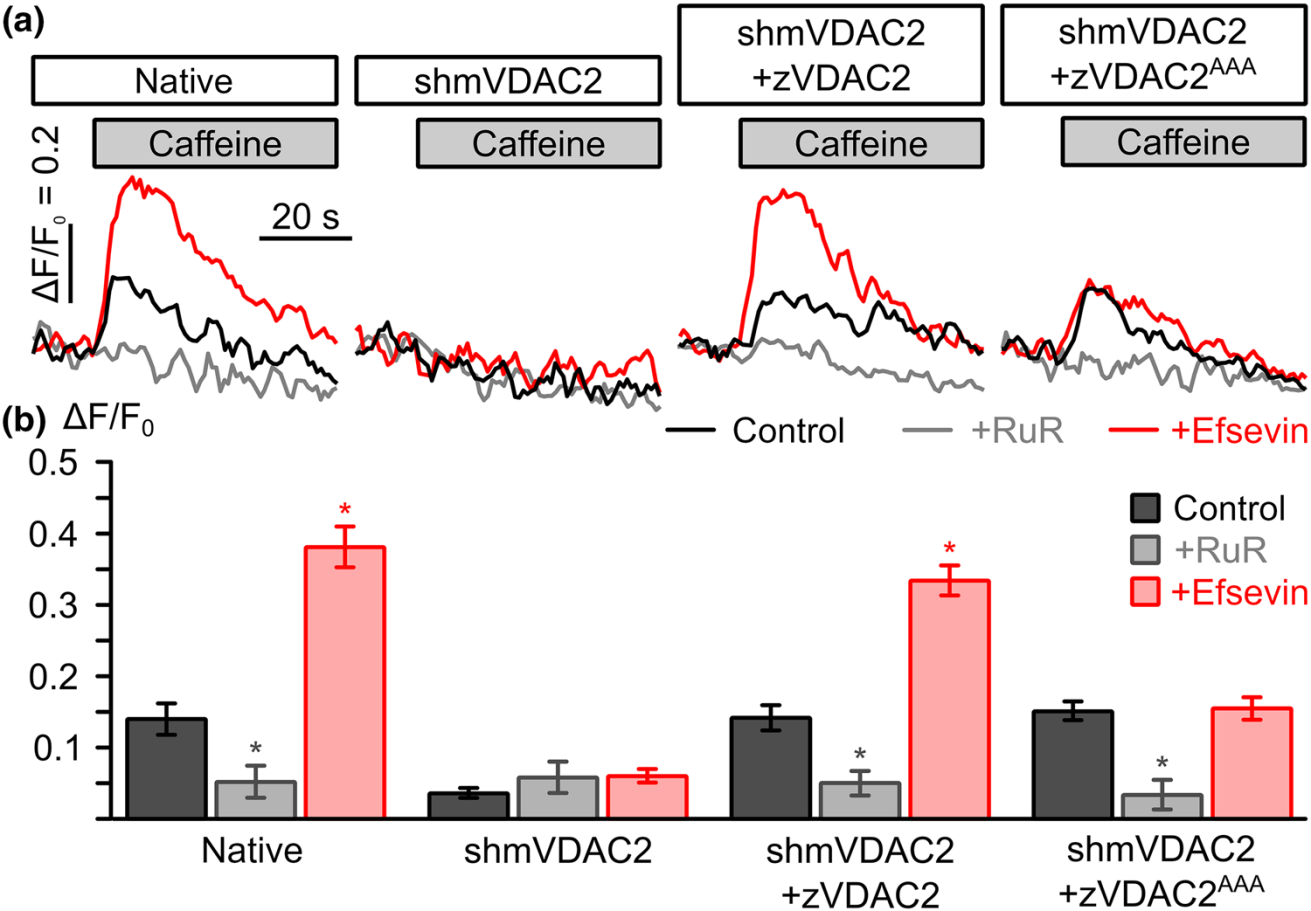
Sarcoplasmic reticulum (SR)‐mitochondria Ca^2+^ transfer upon heterologous expression of zVDAC2 and zVDAC2^AAA^ in HL‐1 cardiomyocytes. (a) Representative recordings of mitochondrial Ca^2+^ upon application of 10‐mM caffeine to induce SR calcium release from permeabilized HL‐1 cardiomyocytes. Traces from control conditions (black), recordings in the presence of 10‐μM ruthenium red to block mitochondrial Ca^2+^ uptake (RuR, grey) and in the presence of 10‐μM efsevin (red) are shown for native HL‐1 cells (native), cells transduced with shRNA targeting the endogenous mouse mVDAC2 (shmVDAC2), and cells overexpressing zVDAC2 and zVDAC2^AAA^, respectively. (b) Statistical analysis of SR‐mitochondria Ca^2+^ transfer experiments. While native HL‐1 cardiomyocytes showed an efsevin‐sensitive uptake of Ca^2+^ into mitochondria (*n* = 21 for control, *n* = 7 for RuR, and *n* = 18 for efsevin), this uptake was abolished upon knock‐down of the endogenous mVDAC2 (shmVDAC2, *n* = 24 for control, *n* = 8 for RuR, *n* = 15 for efsevin). Subsequent heterologous expression of zVDAC2 (shmVDAC2, *n* = 21 for control, *n* = 7 for RuR, *n* = 15 for efsevin) and zVDAC2^AAA^ (shmVDAC2^AAA^, *n* = 18 for control, *n* = 6 for RuR, *n* = 18 for efsevin) revealed restoration of SR‐mitochondria Ca^2+^ transfer. However, only zVDAC2 but not zVDAC2^AAA^ was sensitive to efsevin (Kruskal–Wallis test with Dunn's post hoc test)

To evaluate if the observed efsevin‐induced electrophysiological changes can explain the enhanced mitochondrial Ca^2+^ uptake observed previously for HeLa cells and HL‐1 cardiomyocytes (Schweitzer et al., 2017; Shimizu et al., 2015), we developed a heterologous expression system for zVDAC2. To this aim, we created a stable HL‐1 cardiomyocyte line in which the endogenous mouse VDAC2 (mVDAC2) was knocked down by stable expression of shRNA (Subedi et al., 2011; Figure S4) and overexpressed shRNA‐insensitive zVDAC2 constructs by lentiviral transduction. Ca^2+^ uptake into mitochondria upon caffeine‐induced Ca^2+^ release from the sarcoplasmic reticulum (SR) was then measured in permeabilized Rhod‐2 stained cells (Figure 5). In line with previous experiments, knock‐down of the endogenous mVDAC2 eliminated transfer of Ca^2+^ from the sarcoplasmic reticulum into mitochondria (Subedi et al., 2011). While wild‐type HL‐1 cardiomyocytes displayed maximum ΔF/F_0_ values of 0.14 ± 0.02 that were enhanced to 0.38 ± 0.03 by 10‐μM efsevin, shRNA‐expressing cells displayed ΔF/F_0 max_ values of 0.04 ± 0.03, which were indistinguishable from those obtained from cells treated with the mitochondrial Ca^2+^ uptake blocker ruthenium red (RuR; ΔF/F_0 max_ = 0.06 ± 0.02) or efsevin (ΔF/F_0 max_ = 0.06 ± 0.01, not significant). Though lack of VDAC2 was previously described to induce apoptosis and we can thus not rule out downstream effects induced by mVDAC2 knock down in these cells, the shmVDAC2 cell line was stable for the time of our experiments and thus suitable for heterologous overexpression experiments. Overexpression of zVDAC2 completely restored sarcoplasmic reticulum‐mitochondria Ca^2+^ transfer to ΔF/F_0 max_ = 0.14 ± 0.02, which was sensitive to modulation by efsevin (ΔF/F_0 max_ = 0.34 ± 0.02). Strikingly, zVDAC2^AAA^ likewise restored mitochondrial Ca^2+^ uptake to levels indistinguishable from wild‐type zVDAC2 (ΔF/F_0 max_ = 0.15 ± 0.02) but was insensitive to treatment with efsevin (ΔF/F_0 max_ = 0.16 ± 0.02). These data strongly indicate that the efsevin induced changes in zVDAC2 electrophysiology account for the enhanced mitochondrial Ca^2+^ uptake in cardiomyocytes.

## 4 DISCUSSION

Here, we describe the efsevin–zVDAC2 interaction at a biophysical and structural level. Efsevin was previously shown to enhance mitochondrial Ca^2+^ uptake and to be a powerful modulator of cardiac rhythmicity and a potent suppressor of cardiac arrhythmia. Efsevin suppresses arrhythmogenesis in both, models for Ca^2+^ overload and models for inherited arrhythmias like catecholaminergic polymorphic ventricular tachycardia. In a murine model for CPVT, efsevin significantly reduced episodes of ventricular tachycardia, while no adverse effects of efsevin were observed after up to 8 days of continuous treatment. VDAC2, which was previously described as an essential component of the mitochondrial Ca^2+^ uptake machinery of the heart (Subedi et al., 2011), was identified as the primary target of efsevin (Shimizu et al., 2015). These findings make VDAC2‐mediated mitochondrial Ca^2+^ uptake a promising therapeutic target for the development of a novel class of antiarrhythmic drugs with efsevin as a lead candidate. However, efsevin was identified in a chemical screen using 168 newly synthesized diversity‐oriented compounds (Shimizu et al., 2015) without further compound optimization. In our HL‐1‐based sarcoplasmic reticulum‐mitochondria Ca^2+^ transfer assay, efsevin showed a half‐maximal activity at 2.2 μM (Figure S5A). Preliminary results on efsevin's pharmacokinetics show that it is hydrolysed rapidly in liver microsomes (Figure S5B). Furthermore, binding of efsevin to other targets, including VDAC isoforms 1 and 3, was never tested. Thus, its unknown selectivity, the low stability of efsevin and the relatively high EC_50_ value indicate the need for chemical optimization of the compound before further preclinical and clinical studies are performed. In this study, we present a molecular model of the efsevin binding site on zVDAC2 and thereby provide a basis for structure‐based drug optimization in future experiments.

When discussing VDAC2 as a potential drug target for cardiac arrhythmia, it should be noted that the transfer of Ca^2+^ from the sarcoplasmic reticulum into mitochondria is most likely a specialized role of VDAC2 in cardiomyocytes, presumably accomplished by a functional or even physical coupling to the ryanodine receptor (Min et al., 2012; Shimizu et al., 2015; Subedi et al., 2011) and might be less relevant or maybe accomplished by other VDAC isoforms in other cell types. In fact, VDAC1 was previously reported to promote Ca^2+^ transfer from the endoplasmic reticulum into mitochondria in non‐excitable cells through coupling to the IP_3_ receptor (De Stefani et al., 2012; Rapizzi et al., 2002; Szabadkai et al., 2006). It is thus conceivable that despite the ubiquitous expression of VDACs in the body, efsevin‐mediated effects are most pronounced or even limited to cardiomyocytes due to the specialized role of VDAC2 in this tissue. This effect might also explain the lack of major side effects that were observed previously in translational models (Schweitzer et al., 2017). However, future studies are needed to evaluate the role of the efsevin‐mediated effects on other VDAC2‐mediated effects like apoptosis or regulation of bioenergetics (for a review, see Naghdi & Hajnóczky, 2016). Furthermore, although VDAC2 was identified as the primary target of efsevin and efsevin‐induced antiarrhythmic effects were shown to depend on VDAC2 (Shimizu et al., 2015), interaction of efsevin with other VDAC isoforms were never investigated. Given a high sequence conservation of the residues involved in VDAC2 binding identified in this study, it is conceivable that efsevin binds to VDAC isoforms 1 and 3. This effect might be less relevant during short‐term treatment of mice and fish but might have long‐term impacts on, for example, bioenergetics and thus needs to be investigated in future studies.

Using computational docking on the crystal structure of zVDAC2, we identified a binding pocket formed by the inner channel wall and the N‐terminal helix. The putative binding site was confirmed by replacing three prominent residues inside the pocket by alanines which resulted in insensitivity of the channel to efsevin in lipid bilayers. It is of note that we used a C‐terminal His_6_‐tag for purification of the channel for subsequent bilayer experiments which is not present in the structure used for computational docking. It is thus conceivable that the His_6_‐tag interferes with efsevin and promotes effects seen in bilayer experiments. However, the His_6_‐tag is located approximately 25 Å away from the proposed binding site on the opposed mouth of the channel making interference unlikely. Furthermore, we evaluated the proposed binding site by heterologous expression of zVDAC2 without the C‐terminal His_6_‐tag in HL‐1 cardiomyocytes and found that efsevin sensitivity of the channel was again promoted through the proposed binding site. To prove translatability of the results obtained from zVDAC2, we created a homology model of hVDAC2 using zVDAC2 as a template and found that the lowest bind energy was calculated in the same pocket in eight out of 10 individual docking experiments. The mammalian channel includes additional 11 amino acids on the N‐terminus of the channel. Unfortunately, no homologous structures of this additional sequence have been resolved so far, and coordinates for homology modelling are missing. It is thus again conceivable that the additional N‐terminus in mammalian channels could interfere with efsevin binding. However, in HL‐1 cardiomyocytes, we observe very similar effects of efsevin on native cells expressing the endogenous mVDAC2, which includes the N‐terminal extension and heterologously expressed zVDAC2. Although these data do not rule out the possibility that efsevin promotes its effects on the two distinct channels differently, it argues against it.

Our data indicate that efsevin binds to the inner wall of the β‐barrel in close proximity to the N‐terminal α‐helix and thereby affects channel conductance and gating. Although still not fully resolved, various models were proposed to explain gating of VDACs. Almost all models include a role of the N‐terminal α‐helix ranging from relatively small movements of the helix inside the barrel (Mertins et al., 2012; Shuvo, Ferens, & Court, 2016) to a large movement that place the helix outside of the barrel, which then collapses to induce channel closure (Choudhary et al., 2010; Zachariae et al., 2012). Furthermore, a fixation of the helix inside the pore through disulfate bridges was reported as a possible mechanism to regulate channel gating by redox sensing (Okazaki et al., 2015; Reina et al., 2016). Our data identified a binding pocket for efsevin located in a groove between the N‐terminal α‐helix and the inner channel wall. In all our predicted binding conformations, efsevin is bound to the channel wall through hydrogen bonds provided by N19, N207, R218, K236 or N238 plus additional hydrophobic interactions and interacts with a binding pocket located on the hinge of the α‐helix. Interestingly, one of the residues in the channel wall, K236, was previously reported to form a hydrogen bond with F18 in the α‐helix (Ujwal et al., 2008), a residue that interacts with efsevin upon efsevin binding. It is thus conceivable that efsevin interferes with the interaction between the barrel and the helix in zVDAC2 and consequently affects the movement of the α‐helix required to modulate the channel. However, it should be noted that the binding of efsevin might induce a conformational change in the channel, which is not considered in our docking model. Further experimental and computational investigations, like molecular dynamics simulations and determination of the ligand‐bound VDAC2 structure are thus needed to clarify the exact mode of gating and ion conduction through the channel in the presence and absence of efsevin.

Using an electrophysiological approach, we found efsevin‐promoted gating of zVDAC2. Already at low potentials, the channel preferentially gates into the two closed states and only rarely returns to the open state. We demonstrate that the efsevin‐induced low conducting states display less anion selectivity compared to the open state which translates into a higher Ca^2+^ flux and consequently into higher Ca^2+^ uptake into mitochondria in cardiomyocytes. This is in line with previous experimental (Tan & Colombini, 2007) and computational (Zachariae et al., 2012) studies, which have shown the closed states of VDAC to be more cation‐selective. The observed shift from the open to the closed states is especially relevant at low potentials ranging from −40 to +40 mV. Though it is still under debate whether a membrane potential difference exists over the outer mitochondrial membrane, modelling data suggest a potential of approximately −20 to −27 mV (Lemeshko, 2006) and a study based on pH measurements suggests a potential of approximately −40 mV (Porcelli et al., 2005). This most likely explains the enhanced Ca^2+^ uptake into mitochondria seen in the presence of efsevin. The data presented in this study are thus in agreement with the concept of increased Ca^2+^ flux upon channel closure.

In this study, we present a binding pocket in zVDAC2 and an associated biophysical mechanism, namely, a modification of voltage gating and a shift towards the cation‐selective closed states. VDACs were previously suggested as promising drug targets mainly because of their prominent role in apoptosis (for a review, see Magrì, Reina, & De Pinto, 2018). However, it remained unclear whether the role of VDAC in apoptosis was directly associated with the channel's gating behaviour or rather with a modified interaction with partner proteins such as hexokinase or members of the Bcl‐2 family. In this study, we present evidence that the efsevin‐induced change in gating facilitates transfer of Ca^2+^ from the SR into mitochondria. Interestingly, we never observed an involvement of the drug in apoptosis, neither in this study nor in previous studies (Schweitzer et al., 2017; Shimizu et al., 2015) despite a previously identified isoform‐specific role for VDAC2 in apoptosis through recruitment of Bax (Lauterwasser et al., 2016; Ma et al., 2014). Efsevin could thus serve as a tool to dissect these functions in future studies.

Taken together, we provide functional and structural data that explains the interaction between zVDAC2 and its modulator efsevin on a molecular basis. Our data provide new insights into VDAC2 function as well as a basis for computer‐aided design of optimized compounds that could serve as research compounds and therapeutics for cardiac arrhythmia.

## CONFLICT OF INTEREST

The authors declare no conflicts of interest.

## AUTHOR CONTRIBUTIONS

J.S. and T.G. conceptualized the study and acquired funding. F.W. and J.S. performed bilayer recordings and mitochondrial Ca^2+^ uptake experiments and analysed results. P.G. performed and analysed ion selectivity recordings. R.K. and A.S. performed molecular docking experiments. O.K. and N.J.D. synthesized efsevin. A.N. analysed data. J.S. wrote the manuscript. All authors commented on the manuscript.

## DECLARATION OF TRANSPARENCY AND SCIENTIFIC RIGOUR

This Declaration acknowledges that this paper adheres to the principles for transparent reporting and scientific rigour of preclinical research as stated in the *BJP* guidelines for Design & Analysis, and as recommended by funding agencies, publishers and other organisations engaged with supporting research.

## Supporting information

Figure S1. Cartoon representation of zVDAC2 (pdb: 4bum, light brown) bound to efsevin in (A) top view and (B) side view. The respective best conformations from 10 independent docking experiments are shown.
Figure S2. Cartoon representation of hVDAC2 (created by homology modelling using SWISS‐MODEL with zVDAC2, pdb: 4bum, as template) bound to efsevin in (A) top view and (B) side view. The respective best conformations from 10 independent docking experiments are shown. 8 out of 10 docking experiments revealed binding of efsevin into the same binding pocket as identified for zVDAC2.
Figure S3. Electrophysiological parameters of zVDAC2 compared to zVDAC2^AAA^. No differences could be observed for the conductance‐voltage relationship (A), single channel conductance (B), and open probability (C). (n=9 individual channels for zVDAC2 and 4 individual channels for zVDAC2^AAA^)
Figure S4. Knock‐down of mVDAC2 in HL‐1 cardiomyocytes. (A) Realtime PCR for mVDAC2 on RNA from native HL‐1 cardiomyocytes, a line stably expressing shRNA directed against mVDAC2 (shmVDAC2), and one expressing a scrambled control shRNA (shCtrl) reveals efficient knock‐down of mVDAC2 using shRNA. (B and C) Mitochondrial Ca^2+^ uptake experiments (as described in Figure 5) for HL‐1 cardiomyocytes expressing a scrambled shRNA as a control reveal functional and efsevin‐sensitive SR‐mitochondrial Ca^2+^ transfer comparable to control (Fig. 5). (n=18 for control, n=6 for RuR, n=15 for efsevin, Kruskal–Wallis test with Dunn's post hoc test)
Figure S5. (A) Dose‐dependence of efsevin action on HL‐1 cardiomyocytes. Mitochondrial calcium uptake in response to SR Ca^2+^ release by caffeine was probed in permeabilized HL‐1 cardiomyocytes (left panel). Addition of increasing concentrations of efsevin revealed a dose‐dependent increase in SR‐mitochondria Ca^2+^ transfer with a EC_50_ of 2.2μM (right panel). (B) Stability of efsevin in human liver microsomes. 1μM of efsevin was added to purified human liver microsomes and the reaction was started by addition of NADPH. The remaining concentration of efsevin was determined by LC‐MS/MS. Efsevin was completely hydrolysed within 10 minutes after starting the reaction.

## References

[bph15022-bib-0002] Benjamin, E. J. , Virani, S. S. , Callaway, C. W. , Chamberlain, A. M. , Chang, A. R. , Cheng, S. , … American Heart Association Council on Epidemiology and Prevention Statistics Committee and Stroke Statistics Subcommittee . (2018). Heart disease and stroke statistics—2018 update: A report from the American Heart Association. Circulation, 137, e67–e492. 10.1161/CIR.0000000000000558 29386200

[bph15022-bib-0003] Cheng, E. H. Y. , Sheiko, T. V. , Fisher, J. K. , Craigen, W. J. , & Korsmeyer, S. J. (2003). VDAC2 inhibits BAK activation and mitochondrial apoptosis. Science, 301, 513–517. 10.1126/science.1083995 12881569

[bph15022-bib-0004] Choudhary, O. P. , Ujwal, R. , Kowallis, W. , Coalson, R. , Abramson, J. , & Grabe, M. (2010). The electrostatics of VDAC: Implications for selectivity and gating. Journal of Molecular Biology, 396, 580–592. 10.1016/j.jmb.2009.12.006 20005234 PMC3736979

[bph15022-bib-0005] Claycomb, W. C. , Lanson, N. A. , Stallworth, B. S. , Egeland, D. B. , Delcarpio, J. B. , Bahinski, A. , & Izzo, N. J. (1998). HL‐1 cells: A cardiac muscle cell line that contracts and retains phenotypic characteristics of the adult cardiomyocyte. Proceedings of the National Academy of Sciences of the United States of America, 95, 2979–2984. 10.1073/pnas.95.6.2979 9501201 PMC19680

[bph15022-bib-0006] Colombini, M. (1989). Voltage gating in the mitochondrial channel, VDAC. Journal of Membrane Biology, 111, 103–111. 10.1007/bf01871775 2482359

[bph15022-bib-0007] Curtis, M. J. , Alexander, S. , Cirino, G. , Docherty, J. R. , George, C. H. , Giembycz, M. A. , … Ahluwalia, A. (2018). Experimental design and analysis and their reporting II: Updated and simplified guidance for authors and peer reviewers. British Journal of Pharmacology, 175, 987–993. 10.1111/bph.14153 29520785 PMC5843711

[bph15022-bib-0008] De Stefani, D. , Bononi, A. , Romagnoli, A. , Messina, A. , De Pinto, V. , Pinton, P. , & Rizzuto, R. (2012). VDAC1 selectively transfers apoptotic Ca^2+^ signals to mitochondria. Cell Death and Differentiation, 19, 267–273. 10.1038/cdd.2011.92 21720385 PMC3263501

[bph15022-bib-0009] Ebert, A. M. , Hume, G. L. , Warren, K. S. , Cook, N. P. , Burns, C. G. , Mohideen, M. A. , … Garrity, D. M. (2005). Calcium extrusion is critical for cardiac morphogenesis and rhythm in embryonic zebrafish hearts. Proceedings of the National Academy of Sciences of the United States of America, 102, 17705–17710. 10.1073/pnas.0502683102 16314582 PMC1308882

[bph15022-bib-0010] Guardiani, C. , Magrì, A. , Karachitos, A. , Di Rosa, M. C. , Reina, S. , Bodrenko, I. , … De Pinto, V. (2018). yVDAC2, the second mitochondrial porin isoform of *Saccharomyces cerevisiae* . Biochimica et Biophysica Acta, Bioenergetics, 1859, 270–279. 10.1016/j.bbabio.2018.01.008 29408701

[bph15022-bib-0011] Harding, S. D. , Sharman, J. L. , Faccenda, E. , Southan, C. , Pawson, A. J. , Ireland, S. , … NC‐IUPHAR . (2018). The IUPHAR/BPS Guide to pharmacology in 2018: Updates and expansion to encompass the new guide to immunopharmacology. Nucleic Acids Research, 46, D1091–D1106. 10.1093/nar/gkx1121 29149325 PMC5753190

[bph15022-bib-0012] Henry, C. E. , Xu, Q. , Fan, Y. C. , Martin, T. J. , Belding, L. , Dudding, T. , & Kwon, O. (2014). Hydroxyproline‐derived pseudoenantiomeric [2.2.1] bicyclic phosphines: Asymmetric synthesis of (+)‐ and (−)‐pyrrolines. Journal of the American Chemical Society, 136, 11890–11893. 10.1021/ja505592h 25099350 PMC4151783

[bph15022-bib-0013] Hille, B. (2001). Ion channels of excitable membranes. Sunderland, MA, USA: Sinauer Associates Inc.

[bph15022-bib-8001] Kirichok, Y. , Krapivinsky, G. & Clapham, D. (2004). The mitochondrial calcium uniporter is a highly selective ion channel. Nature, 427, 360–364. 14737170 10.1038/nature02246

[bph15022-bib-0014] Kwan, K. M. , Fujimoto, E. , Grabher, C. , Mangum, B. D. , Hardy, M. E. , Campbell, D. S. , … Chien, C. B. (2007). The Tol2kit: A multisite gateway‐based construction kit for Tol2 transposon transgenesis constructs. Developmental Dynamics, 236, 3088–3099. 10.1002/dvdy.21343 17937395

[bph15022-bib-0015] Langenbacher, A. D. , Dong, Y. , Shu, X. , Choi, J. , Nicoll, D. A. , Goldhaber, J. I. , … Chen, J. N. (2005). Mutation in sodium‐calcium exchanger 1 (NCX1) causes cardiac fibrillation in zebrafish. Proceedings of the National Academy of Sciences of the United States of America, 102, 17699–17704. 10.1073/pnas.0502679102 16314583 PMC1308881

[bph15022-bib-0016] Laskowski, R. A. , & Swindells, M. B. (2011). LigPlot+: Multiple ligand–protein interaction diagrams for drug discovery. Journal of Chemical Information and Modeling, 51, 2778–2786. 10.1021/ci200227u 21919503

[bph15022-bib-0017] Lauterwasser, J. , Todt, F. , Zerbes, R. M. , Nguyen, T. N. , Craigen, W. , Lazarou, M. , … Edlich, F. (2016). The porin VDAC2 is the mitochondrial platform for Bax retrotranslocation. Scientific Reports, 6, 32994 10.1038/srep32994 27620692 PMC5020405

[bph15022-bib-0018] Lemeshko, V. V. (2006). Theoretical evaluation of a possible nature of the outer membrane potential of mitochondria. European Biophysics Journal, 36, 57–66. 10.1007/s00249-006-0101-7 17021806

[bph15022-bib-0019] Lide, D. R. (2006). CRC handbook of chemistry and physics (87th ed.). Boca Raton: CRC Press, Taylor & Francis Group.

[bph15022-bib-0020] Ma, S. B. , Nguyen, T. N. , Tan, I. , Ninnis, R. , Iyer, S. , Stroud, D. A. , … Dewson, G. (2014). Bax targets mitochondria by distinct mechanisms before or during apoptotic cell death: a requirement for VDAC2 or Bak for efficient Bax apoptotic function. Cell Death and Differentiation, 21, 1925–1935. 10.1038/cdd.2014.119 25146925 PMC4227151

[bph15022-bib-0021] Magrì, A. , Reina, S. , & De Pinto, V. (2018). VDAC1 as pharmacological target in cancer and neurodegeneration: Focus on its role in apoptosis. Frontiers in Chemistry, 6, 108. 29682501 10.3389/fchem.2018.00108 PMC5897536

[bph15022-bib-0022] Menzel, V. A. , Cassará, M. C. , Benz, R. , De Pinto, V. , Messina, A. , Cunsolo, V. , … Hinsch, E. (2009). Molecular and functional characterization of VDAC2 purified from mammal spermatozoa. Bioscience Reports, 29, 351–362. 10.1042/BSR20080123 18976238

[bph15022-bib-0023] Mertins, B. , Psakis, G. , Grosse, W. , Back, K. C. , Salisowski, A. , Reiss, P. , … Essen, L. O. (2012). Flexibility of the N‐terminal mVDAC1 segment controls the channel's gating behavior. PLoS ONE, 7, e47938 10.1371/journal.pone.0047938 23110136 PMC3479125

[bph15022-bib-0024] Min, C. K. , Yeom, D. R. , Lee, K.‐E. , Kwon, H.‐K. , Kang, M. , Kim, Y.‐S. , … Kim, D. H. (2012). Coupling of ryanodine receptor 2 and voltage‐dependent anion channel 2 is essential for Ca^2+^ transfer from the sarcoplasmic reticulum to the mitochondria in the heart. The Biochemical Journal, 447, 371–379. 10.1042/BJ20120705 22867515

[bph15022-bib-0025] Morris, G. M. , Huey, R. , Lindstrom, W. , Sanner, M. F. , Belew, R. K. , Goodsell, D. S. , & Olson, A. J. (2009). AutoDock4 and AutoDockTools4: Automated docking with selective receptor flexibility. Journal of Computational Chemistry, 30, 2785–2791. 10.1002/jcc.21256 19399780 PMC2760638

[bph15022-bib-0026] Naghdi, S. , & Hajnóczky, G. (2016). VDAC2‐specific cellular functions and the underlying structure. Biochimica et Biophysica Acta, Molecular Cell Research, 1863, 2503–2514. 10.1016/j.bbamcr.2016.04.020 PMC5092071 27116927

[bph15022-bib-0027] Okazaki, M. , Kurabayashi, K. , Asanuma, M. , Saito, Y. , Dodo, K. , & Sodeoka, M. (2015). VDAC3 gating is activated by suppression of disulfide‐bond formation between the N‐terminal region and the bottom of the pore. Biochimica et Biophysica Acta (BBA)‐Biomembranes, 1848, 3188–3196. 26407725 10.1016/j.bbamem.2015.09.017

[bph15022-bib-0028] Porcelli, A. M. , Ghelli, A. , Zanna, C. , Pinton, P. , Rizzuto, R. , & Rugolo, M. (2005). pH difference across the outer mitochondrial membrane measured with a green fluorescent protein mutant. Biochemical and Biophysical Research Communications, 326, 799–804. 10.1016/j.bbrc.2004.11.105 15607740

[bph15022-bib-0029] Raghavan, A. , Sheiko, T. V. , Graham, B. H. , & Craigen, W. J. (2012). Voltage‐dependant anion channels: Novel insights into isoform function through genetic models. Biochimica et Biophysica Acta‐Biomembranes, 1818, 1477–1485. 10.1016/j.bbamem.2011.10.019 PMC4273737 22051019

[bph15022-bib-0030] Rapizzi, E. , Pinton, P. , Szabadkai, G. , Wieckowski, M. R. , Vandecasteele, G. , Baird, G. , … Rizzuto, R. (2002). Recombinant expression of the voltage‐dependent anion channel enhances the transfer of Ca^2+^ microdomains to mitochondria. The Journal of Cell Biology, 159, 613–624. 10.1083/jcb.200205091 12438411 PMC2173108

[bph15022-bib-0031] Reina, S. , Checchetto, V. , Saletti, R. , Gupta, A. , Chaturvedi, D. , Guardiani, C. , … de Pinto, V. (2016). VDAC3 as a sensor of oxidative state of the intermembrane space of mitochondria: The putative role of cysteine residue modifications. Oncotarget, 7, 2249–2268. 10.18632/oncotarget.6850 26760765 PMC4823033

[bph15022-bib-0032] Rostovtseva, T. K. , Gurnev, P. A. , Chen, M.‐Y. , & Bezrukov, S. M. (2012). Membrane lipid composition regulates tubulin interaction with mitochondrial voltage‐dependent anion channel. The Journal of Biological Chemistry, 287, 29589–29598. 10.1074/jbc.M112.378778 22763701 PMC3436136

[bph15022-bib-0033] Schredelseker, J. , Paz, A. , López, C. J. , Altenbach, C. , Leung, C. S. , Drexler, M. K. , … Abramson, J. (2014). High‐resolution structure and double electron‐electron resonance of the zebrafish voltage dependent anion channel 2 reveal an oligomeric population. The Journal of Biological Chemistry, 289, 12566–12577. 10.1074/jbc.M113.497438 24627492 PMC4007448

[bph15022-bib-0034] Schweitzer, M. K. , Wilting, F. , Sedej, S. , Dreizehnter, L. , Dupper, N. J. , Tian, Q. , … Schredelseker, J. (2017). Suppression of arrhythmia by enhancing mitochondrial Ca^2+^ uptake in catecholaminergic ventricular tachycardia models. JACC Basic to Translational Science, 2, 737–746. 10.1016/j.jacbts.2017.06.008 29354781 PMC5774336

[bph15022-bib-0035] Shimizu, H. , Schredelseker, J. , Huang, J. , Lu, K. , Naghdi, S. , Lu, F. , … Chen, J. N. (2015). Mitochondrial Ca^2+^ uptake by the voltage‐dependent anion channel 2 regulates cardiac rhythmicity. eLife, 4 10.7554/eLife.04801 PMC4293673 25588501

[bph15022-bib-0036] Shuvo, S. R. , Ferens, F. G. , & Court, D. A. (2016). The N‐terminus of VDAC: Structure, mutational analysis, and a potential role in regulating barrel shape. Biochimica et Biophysica Acta‐Biomembranes, 1858, 1350–1361. 10.1016/j.bbamem.2016.03.017 26997586

[bph15022-bib-0037] Subedi, K. P. , Kim, J.‐C. , Kang, M. , Son, M.‐J. , Kim, Y.‐S. , & Woo, S.‐H. (2011). Voltage‐dependent anion channel 2 modulates resting Ca^2+^ sparks, but not action potential‐induced Ca^2+^ signaling in cardiac myocytes. Cell Calcium, 49, 136–143. 10.1016/j.ceca.2010.12.004 21241999

[bph15022-bib-0038] Szabadkai, G. , Bianchi, K. , Várnai, P. , De Stefani, D. , Wieckowski, M. R. , Cavagna, D. , … Rizzuto, R. (2006). Chaperone‐mediated coupling of endoplasmic reticulum and mitochondrial Ca^2+^ channels. The Journal of Cell Biology, 175, 901–911. 10.1083/jcb.200608073 17178908 PMC2064700

[bph15022-bib-0039] Tan, W. , & Colombini, M. (2007). VDAC closure increases calcium ion flux. Biochimica et Biophysica Acta, 1768, 2510–2515. 10.1016/j.bbamem.2007.06.002 17617374 PMC2220155

[bph15022-bib-0040] Trott, O. , & Olson, A. J. (2010). AutoDock Vina: Improving the speed and accuracy of docking with a new scoring function, efficient optimization, and multithreading. Journal of Computational Chemistry, 31, 455–461. 10.1002/jcc.21334 19499576 PMC3041641

[bph15022-bib-0041] Ujwal, R. , & Abramson, J. (2012). High‐throughput crystallization of membrane proteins using the lipidic bicelle method. Journal of Visualized Experiments, 59, e3383. 10.3791/3383 PMC3369771 22257923

[bph15022-bib-0042] Ujwal, R. , Cascio, D. , Colletier, J.‐P. , Faham, S. , Zhang, J. , Toro, L. , … Abramson, J. (2008). The crystal structure of mouse VDAC1 at 2.3 Å resolution reveals mechanistic insights into metabolite gating. Proceedings of the National Academy of Sciences of the United States of America, 105, 17742–17747. 10.1073/pnas.0809634105 18988731 PMC2584669

[bph15022-bib-0043] Waterhouse, A. , Bertoni, M. , Bienert, S. , Studer, G. , Tauriello, G. , Gumienny, R. , … Schwede, T. (2018). SWISS‐MODEL: Homology modelling of protein structures and complexes. Nucleic Acids Research, 46, W296–W303. 10.1093/nar/gky427 29788355 PMC6030848

[bph15022-bib-0044] Zachariae, U. , Schneider, R. , Briones, R. , Gattin, Z. , Demers, J.‐P. , Giller, K. , … Lange, A. (2012). β‐barrel mobility underlies closure of the voltage‐dependent anion channel. Structure, 20, 1540–1549. 10.1016/j.str.2012.06.015 22841291 PMC5650048

