# Supplementary material for: The antiarrhythmic compound efsevin directly modulates voltage‐dependent anion channel 2 by binding to its inner wall and enhancing mitochondrial Ca2+ uptake
Source: Br J Pharmacol. 2020 Mar 25;177(13):2947–58. doi: 10.1111/bph.15022 (PMC7279994; doi:10.1111/bph.15022)
Supplement: Supplementary file 1 — Figure S1. Cartoon representation of zVDAC2 (pdb: 4bum, light brown) bound to efsevin in (A) top view and (B) side view. The respective best conformations from 10 independent docking experiments are shown. Figure S2. Cartoon representation of hVDAC2 (created by homology modelling using SWISS‐MODEL with zVDAC2, pdb: 4bum, as template) bound to efsevin in (A) top view and (B) side view. The respective best conformations from 10 independent docking experiments are shown. 8 out of 10 docking experiments revealed binding of efsevin into the same binding pocket as identified for zVDAC2. Figure S3. Electrophysiological parameters of zVDAC2 compared to zVDAC2AAA. No differences could be observed for the conductance‐voltage relationship (A), single channel conductance (B), and open probability (C). (n=9 individual channels for zVDAC2 and 4 individual channels for zVDAC2AAA) Figure S4. Knock‐down of mVDAC2 in HL‐1 cardiomyocytes. (A) Realtime PCR for mVDAC2 on RNA from native HL‐1 cardiomyocytes, a line stably expressing shRNA directed against mVDAC2 (shmVDAC2), and one expressing a scrambled control shRNA (shCtrl) reveals efficient knock‐down of mVDAC2 using shRNA. (B and C) Mitochondrial Ca2+ uptake experiments (as described in Figure 5) for HL‐1 cardiomyocytes expressing a scrambled shRNA as a control reveal functional and efsevin‐sensitive SR‐mitochondrial Ca2+ transfer comparable to control (Fig. 5). (n=18 for control, n=6 for RuR, n=15 for efsevin, Kruskal–Wallis test with Dunn's post hoc test) Figure S5. (A) Dose‐dependence of efsevin action on HL‐1 cardiomyocytes. Mitochondrial calcium uptake in response to SR Ca2+ release by caffeine was probed in permeabilized HL‐1 cardiomyocytes (left panel). Addition of increasing concentrations of efsevin revealed a dose‐dependent increase in SR‐mitochondria Ca2+ transfer with a EC50 of 2.2μM (right panel). (B) Stability of efsevin in human liver microsomes. 1μM of efsevin was added to purified human liver microsomes and the reac [file BPH-177-2947-s001.pdf]

SUPPORTING INFORMATION

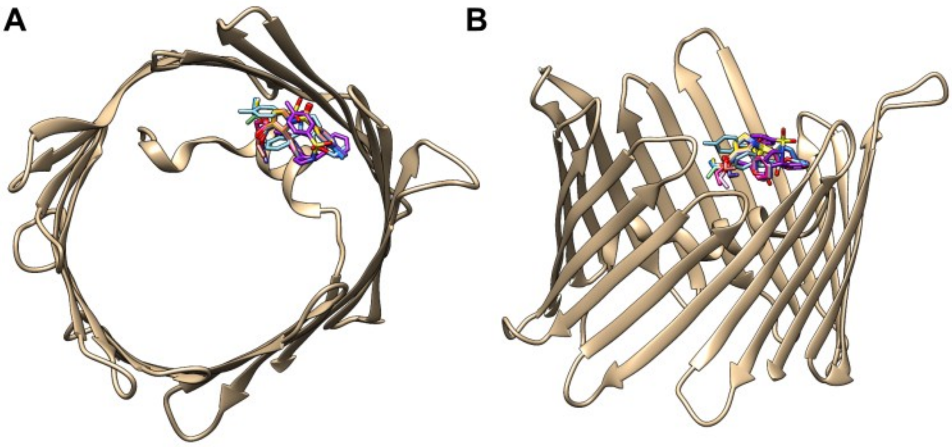

Supporting Information Figure 1. Cartoon representation of zVDAC2 (pdb: 4bum, light brown) bound to efsevin in (A) top view and (B) side view. The respective best conformations from 10 independent docking experiments are shown.

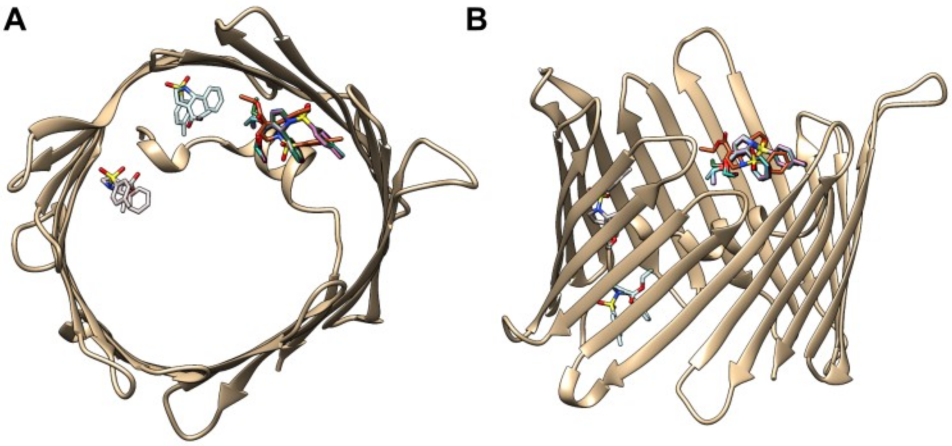

Supporting Information Figure 2. Cartoon representation of hVDAC2 (created by homology modelling using SWISS-MODEL with zVDAC2, pdb: 4bum, as template) bound to efsevin in (A) top view and (B) side view. The respective best conformations from 10 independent docking experiments are shown. 8 out of 10 docking experiments revealed binding of efsevin into the same binding pocket as identified for zVDAC2.

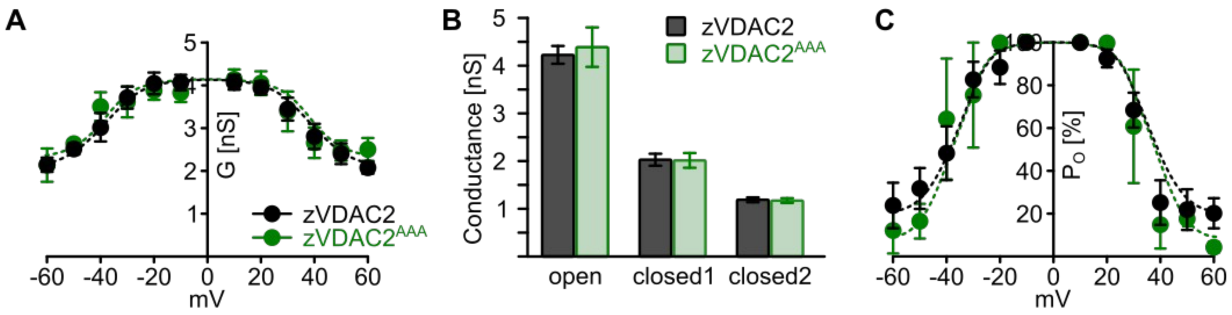

Supporting Information Figure 3. Electrophysiological parameters of zVDAC2 compared to zVDAC2^{AAA}. No differences could be observed for the conductance-voltage relationship (A), single channel conductance (B), and open probability (C). (n=9 individual channels for zVDAC2 and 4 individual channels for zVDAC2^{AAA})

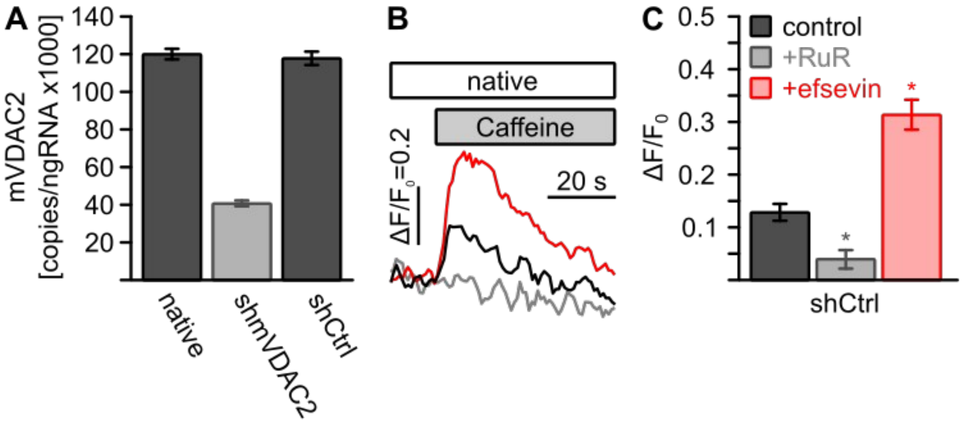

Supporting Information Figure 4. Knock-down of mVDAC2 in HL-1 cardiomyocytes. (A) Real-time PCR for mVDAC2 on RNA from native HL-1 cardiomyocytes, a line stably expressing shRNA directed against mVDAC2 (shmVDAC2), and one expressing a scrambled control shRNA (shCtrl) reveals efficient knock-down of mVDAC2 using shRNA. (B and C) Mitochondrial Ca^{2+} uptake experiments (as described in Figure 5) for HL-1 cardiomyocytes expressing a scrambled shRNA as a control reveal functional and efsevin-sensitive SR-mitochondrial Ca^{2+} transfer comparable to control (Fig. 5). (n=18 for control, n=6 for RuR, n=15 for efsevin, Kruskal-Wallis test with Dunn's post hoc test)

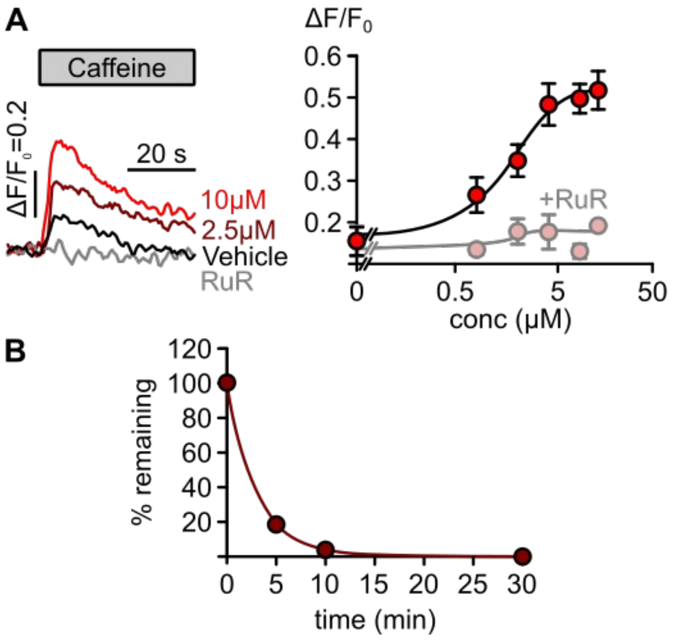

Supporting Information Figure 5. (A) Dose-dependence of efsevin action on HL-1 cardiomyocytes. Mitochondrial calcium uptake in response to SR Ca^{2+} release by caffeine was probed in permeabilized HL-1 cardiomyocytes (left panel). Addition of increasing concentrations of efsevin revealed a dose-dependent increase in SR-mitochondria Ca^{2+} transfer with a EC_{50} of 2.2µM (right panel). (B) Stability of efsevin in human liver microsomes. 1µM of efsevin was added to purified human liver microsomes and the reaction was started by addition of NADPH. The remaining concentration of efsevin was determined by LC-MS/MS. Efsevin was completely hydrolysed within 10 minutes after starting the reaction.
